# Hybrid VLC-RF Channel Estimation for GFDM Wireless Sensor Networks Using Tree-Based Regressor

**DOI:** 10.3390/s25133906

**Published:** 2025-06-23

**Authors:** Azam Isam Aladwani, Tarik Adnan Almohamad, Abdullah Talha Sözer, İsmail Rakıp Karaş

**Affiliations:** 1 Electrical and Electronics Engineering Department, Faculty of Engineering, Karabuk University, Karabuk 78050, Türkiye; azzam.esam@ntu.edu.iq (A.I.A.); talhasozer@karabuk.edu.tr (A.T.S.); 2 Computer Engineering Department, Faculty of Engineering, Karabuk University, Karabuk 78050, Türkiye; ismail.karas@karabuk.edu.tr

**Keywords:** wireless sensor networks (WSNs), generalized frequency division multiplexing (GFDM), tree-based machine learning, channel estimation, visible light communication (VLC), radio frequency (RF), hybrid channel

## Abstract

This paper proposes a tree-based regression model for hybrid channel estimation in wireless sensor networks (WSNs) in generalized frequency division multiplexing (GFDM) over both visible light communication (VLC) and radio frequency (RF) links. The hybrid channel incorporates both additive white Gaussian noise (AWGN) and Rayleigh fading to mimic realistic environments. Traditional estimators, such as MMSE and LMMSE, often underperform in such heterogeneous and nonlinear conditions due to their analytical rigidity. To overcome these limitations, we introduce a data-driven approach using a decision tree regressor trained on 18,000 signal samples across 36 SNR levels. Simulation results show that support vector machine (SVM) achieved 91.34% accuracy and a BER of 0.0866 at 10 dB, as well as 96.77% accuracy with a BER of 0.0323 at 30 dB. Random forest achieved 91.01% accuracy and a BER of 0.0899 at 10 dB, as well as 97.88% accuracy with a BER of 0.0212 at 30 dB. The proposed tree model attained 90.83% and 97.63% accuracy with BERs of 0.0917 and 0.0237, respectively, at the corresponding SNR values. The distinguishing advantage of the tree model lies in its inference efficiency. It completes predictions on the test dataset in just 45.53 s, making it over three times faster than random forest (140.09 s) and more than four times faster than SVM (189.35 s). This significant reduction in inference time makes the proposed tree model particularly well suited for real-time and resource-constrained WSN scenarios, where fast and efficient estimation is often more critical than marginal gains in accuracy. The results also highlight a trade-off, where the tree model provides sub-optimal predictive performance while significantly reducing computational overhead, making it an attractive choice for low-power and latency-sensitive wireless systems.

## 1. Introduction

Wireless sensor networks (WSNs) represent a significant advancement in the deployment of intelligent systems across multiple domains, including smart cities, environmental monitoring, healthcare, and industrial automation. These networks consist of distributed sensor nodes that are energy-constrained and operate in dynamic settings, necessitating communication protocols that prioritize energy efficiency, low latency, and resilience to environmental interference [1]. Traditional radio frequency (RF) communication methods have proven effective; however, they face major issues, such as spectrum congestion, electromagnetic interference, and security vulnerabilities, thus prompting research into alternative communication methods [1,2].

Recently, visible light communication (VLC) has been recognized as a promising alternative owing to its significant advantages, including extensive unlicensed bandwidth and immunity to electromagnetic interference [2,3]. Nevertheless, VLC is intrinsically limited by its reliance on line-of-sight conditions and changes in ambient lighting, leading researchers to develop hybrid VLC-RF systems that combine the strengths of both communication methods. These hybrid systems enhance coverage and robustness while improving spectral efficiency, making them suitable for next-generation WSN applications [4].

Despite these advantages, hybrid VLC-RF systems present new technical challenges, particularly in channel estimation. The deterministic nature of VLC channels, juxtaposed with the stochastic characteristics of RF channels, complicates traditional estimation techniques as RF channels are often subject to multipath propagation effects. Advanced methods further complicate the scenario with inherent non-orthogonal characteristics that lead to inter-symbol interference (ISI), such as generalized frequency division multiplexing (GFDM) [4]. Consequently, conventional means of channel estimation, including Minimum Mean Square Error (MMSE) and Linear MMSE (LMMSE) techniques, tend to perform suboptimally in these mixed environments, necessitating the adoption of more sophisticated solutions [5].

In light of these challenges, there is a growing trend toward leveraging machine learning (ML) algorithms to enhance channel estimation methods in hybrid systems. Recent studies have demonstrated the viability of data-driven approaches, particularly those employing deep learning architectures designed to learn channel characteristics directly from empirical data without rigid model assumptions [6]. Tree-based models, such as decision trees and random forests, have emerged as practical options due to their minimal computational overhead, making them ideally suited for resource-constrained environments [7,8,9]. This trend highlights a critical shift toward integrating ML strategies not only for channel estimation, but also for various roles in optimizing the performance and efficiency of WSNs.

**Figure 1:**
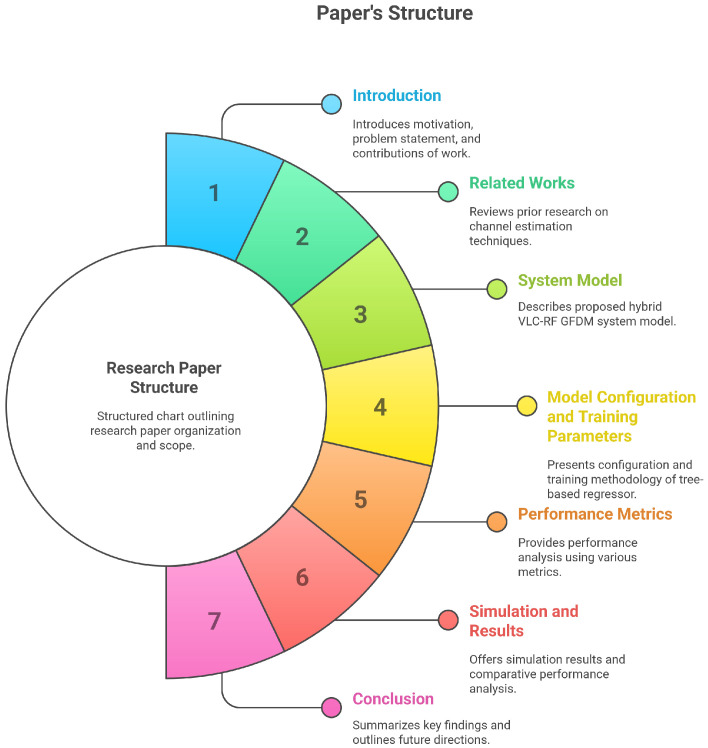
Outline of the entire manuscript.

In fact, WSNs stand at the crossroads of advancing communication technologies and machine learning methods. The integration of VLC and RF in hybrid systems promises substantial improvements over traditional RF communication, although it introduces new challenges in channel estimation that must be effectively addressed through innovative techniques, particularly in the realm of machine learning. Ultimately, these advancements can significantly enhance the capabilities of WSNs in the face of increasing demands from diverse real-time applications. The outline of the paper is shown in Figure 1.

### 1.1. Motivation

Wireless sensor networks (WSNs) are increasingly used in applications that demand real-time responsiveness, low power consumption, and robust communication across diverse environments. Hybrid communication systems combining visible light communication (VLC) and radio frequency (RF) offer a promising solution by leveraging the complementary advantages of both technologies. However, accurately estimating the channel in such systems remains a major challenge, especially under practical indoor conditions influenced by noise and fading.

Traditional estimation methods, such as MMSE and LMMSE, while effective in idealized settings, struggle to adapt to the nonlinear and heterogeneous nature of hybrid VLC-RF channels. Furthermore, while machine learning (ML) models have been explored for channel estimation, most studies either overlook the hybrid VLC-RF scenario or apply complex models that are computationally expensive and poorly suited for the energy and latency constraints of WSNs.

Tree-based machine learning models, especially decision tree regressors, offer a compelling alternative to more complex algorithms due to their simplicity, interpretability, and computational efficiency. Unlike other models, decision trees produce intuitive if–then rule sets, enabling transparent and traceable decision making, which is a critical feature in scenarios requiring fast and explainable results. They are capable of handling both categorical and continuous variables and can effectively model the nonlinear relationships between inputs and outputs. These advantages make them particularly suitable for real-time and resource-constrained applications. [10,11]. Moreover, their potential in hybrid VLC-RF systems, especially under GFDM modulation, remains hugely unexplored. Additionally, a unified evaluation comparing their performance with both traditional and modern ML techniques across a wide SNR range is lacking.

Motivated by these gaps, this study aims to develop and validate a lightweight, scalable, and accurate tree-based channel estimation framework tailored for GFDM-based hybrid VLC-RF WSNs. By emphasizing both predictive performance and inference speed, the proposed approach addresses the critical need for practical, deployable solutions in next-generation wireless networks.

### 1.2. Contributions

In this paper, we propose a novel tree-based regression model for hybrid VLC-RF channel estimation in GFDM-based wireless sensor networks. Our key contributions are summarized as follows:

1. We propose a realistic hybrid communication model combining AWGN-affected VLC and Rayleigh-faded RF channels integrated with GFDM to simulate practical indoor wireless environments.
2. We introduce a decision tree-based regressor to estimate the nonlinear and composite characteristics of hybrid channels by feeding directly received signal vectors, optimizing the model for interpretability and low computational demand.
3. We constructed a large-scale dataset consisting of 18,000 signal samples across 36 distinct SNR levels, ranging from −5 dB to 30 dB, to train and test our model under diverse noise conditions.
4. We benchmarked the proposed model against traditional estimators (MMSE and LMMSE) and modern ML approaches (random forest, support vector machines, and linear regression) using performance metrics, including the Bit Error Rate (BER), accuracy, precision, recall, F1-score, and inference time.
5. We validated the model for real-time deployment by demonstrating that the proposed tree model achieved an accuracy of 90.83% with a BER of 0.0917 at 10 dB, as well as 97.63% with a BER of 0.0237 at 30 dB, while maintaining a practical inference time of approximately 45.53 s for the entire test dataset. In comparison, the random forest model achieved 91.01% accuracy with a BER of 0.0899 at 10 dB, as well as 97.88% accuracy with a BER of 0.0212 at 30 dB, requiring 140.09 s (three times longer than the proposed model) to complete the same task. Similarly, the SVM model achieved 91.34% accuracy with a BER of 0.0866 at 10 dB, as well as 96.77% accuracy with a BER of 0.0323 at 30 dB, but it took 189.35 s (over four times the inference time of the proposed model). This shows that our proposed tree model offers a competitive trade-off between accuracy and inference time, making it a better choice for time-sensitive and low-power WSN applications.

### 1.3. Novelty

The novelty of this work lies in its integrated and lightweight approach to hybrid VLC-RF channel estimation within GFDM-based wireless sensor networks (WSNs). Unlike prior studies that either focused on individual RF or VLC domains or adopted computationally intensive models, our approach uniquely combines the following:

1. We applied and benchmarked a tree decision-based regressor for hybrid VLC-RF channel estimation under GFDM modulation, representing a novel application of this method within this context to the best of our knowledge.
2. We bridged the performance–efficiency gap by achieving near-optimal regression accuracy comparable to complex approaches (e.g., random forest) while significantly reducing inference time, making the proposed work feasible for the latency-sensitive, low-power applications common in WSNs.
3. To support real-time use, the model is built for low-latency inference. After being trained offline, the tree-based model makes fast predictions since it does not require iterative processing. This allows it to meet real-time channel estimation demands, especially when it is further optimized using pre-computed lookup tables.
4. Moreover, the model is optimized for lightweight deployment, considering the limited computational capabilities of typical WSN nodes. The training process is conducted offline on more capable hardware, while inference is carried out on the sensor nodes.
5. We conducted a detailed performance evaluation using 18,000 samples across 36 SNR levels, and we compared our approach with traditional estimators (MMSE and LMMSE) and various machine learning models (SVM, RF, and linear regression).

This combination of hybrid modeling, algorithmic efficiency, deployment practicality, and wide-range benchmarking offers a fresh and practical perspective on deploying ML-based estimators in next-generation WSNs.

## 2. Related Work

Channel estimation in wireless sensor networks (WSNs) has been extensively explored using various methods, including traditional, machine learning (ML), and deep learning (DL) approaches. Each offer distinct benefits but also face specific challenges, particularly in hybrid visible light communication–radio frequency (VLC-RF) environments.

A distributed blind equalization method was proposed in [12], where local outputs are optimized based on channel quality, offering resilience to nonlinear distortions. Building on this, Chi et al. [13] introduced a signal-power-based weight combination rule to further refine blind equalization performance. Despite these improvements, both approaches assume homogeneous signal types and centralized learning, limiting their applicability in heterogeneous VLC-RF WSNs.

For low-power environments, Xie et al. [14] developed a simplified log-likelihood ratio (LLR) estimation under Rayleigh fading. Their approach is computationally efficient but remains narrowly tailored to specific fading and modulation types, restricting its generalization to broader hybrid channel scenarios.

To address mobile jamming, Darsena et al. [15] proposed a blind channel estimation technique for UAV-assisted WSNs. While effective in dynamic aerial scenarios, the method’s reliance on UAV mobility and scenario-specific configurations reduces its suitability for static WSN deployments.

A channel access mechanism was presented in [16] to enhance throughput and fairness. This was extended in a follow-up study [17], where Tauseef et al. employed a game-theoretic fairness model to optimize resource allocation. Although these methods successfully improve access efficiency, they primarily address medium access control rather than the precision of channel estimation under nonlinear channel behaviors.

Khokhar et al. [18] introduced a fractional-order diffusion-based estimation approach, which theoretically accelerates convergence. However, its high computational demands make it less practical for embedded WSN nodes with limited resources.

In [19], Zhu and Ding proposed a variance-based cooperative estimation scheme that dynamically triggers updates based on channel variations, improving energy efficiency. Nevertheless, their method targets RF-only systems and lacks responsiveness in dual-modality hybrid networks.

More recently, Yu et al. [20] applied Kalman and extended Kalman filtering for RIS-assisted channel tracking under pilot contamination and hardware impairments. While effective under mobility, RIS-based solutions still face deployment challenges, particularly in cost-sensitive WSN scenarios.

With the increasing complexity of channel behaviors, ML-based methods have emerged to address nonlinear estimation through data-driven approaches. Recent ML efforts also include shallow ANN architectures for signal-dependent noise mitigation in VLC, targeting low-complexity channel refinement [21]. In [22], two ANN models were proposed for VLC-based spatial modulation under mobility and random receiver orientation, outperforming traditional interpolation methods in CSI prediction. A decision tree-based interference mitigation scheme in [9] was proposed for lightweight WSN deployments. While demonstrating resource efficiency, this method remains confined to RF systems and does not incorporate VLC characteristics.

An unsupervised learning model for VLC-based environmental monitoring was introduced by Ilter et al. [23], capturing spatial characteristics effectively, though limited to static object detection. In a related work, Ilter et al. [24] employed a random forest approach for VLC-based object classification. Although accurate, this approach focuses primarily on semantic interpretation rather than physical-layer channel estimation.

ML techniques were also applied to ATSC 3.0 systems for dynamic vehicular environments by Liu et al. [25], offering strong adaptive capabilities. However, their method demands high-speed computational resources, which are unsuitable for constrained WSN platforms. Gül et al. [26] utilized RF fingerprinting for interference-resilient industrial IoT networks, enhancing security, yet leaving real-time hybrid VLC-RF estimation unaddressed.

In [27], Ahmad and Hussain implemented hybrid random forest and deep learning models for urban vehicular propagation modeling, achieving high accuracy but requiring complex model orchestration. Similarly, Lai [28] applied random forests to underwater optical demodulation. Although effective for nonlinear optical environments, the method is contextually restricted to underwater scenarios.

Saleh et al. [29] provided a comprehensive review of ML and DL methods aimed at enhancing WSN security. While highlighting the intersection between channel estimation and attack resilience, the review focused primarily on security aspects rather than on hybrid estimation adaptability.

In [30], Zha et al. extended support vector regression (SVR) to VLC systems by introducing a time-delay twin SVR model, improving the predistortion accuracy for LED nonlinearities, though remaining limited to optical domains. Similarly, Sun et al. [31] employed SVMs for signal detection in generalized spatial modulation (GSM) VLC systems, achieving improved BER performance at lower complexity compared to maximum likelihood detection; however, their focus remained on detection rather than channel estimation.

Finally, Zaidi et al. [32] proposed a tapped delay line (TDL) model combined with ML-based estimation for environmental awareness. While promising for hybrid-aware sensing, the current application remains limited to static inference scenarios.

Deep learning techniques have pushed further into complex nonlinear modeling. Naikoti and Chockalingam [33] developed a DNN-based OTFS transceiver capable of addressing delay-Doppler and IQ imbalance issues, though its high processing demands limit practical deployment in WSNs.

In [34], a multitask CNN model was proposed to estimate multiple channel parameters simultaneously in vehicular contexts. Although offering strong generalization capabilities, the model requires significant inference time and large data volumes. Similarly, Huang et al. [35] advocated the use of RNNs for capturing time-variant wireless propagation, which is effective for sequence modeling but challenged by training overhead and memory constraints.

Tian et al. [36] introduced RadioNet, a Transformer-based model designed for radio map prediction in dense urban environments. While it demonstrates strong performance in capturing long-range dependencies and handling multipath effects, the model’s high computational complexity and limited interpretability present challenges for deployment in resource-constrained edge wireless sensor network (WSN) applications. Ma et al. [37] utilized deep learning for sparse channel estimation and hybrid precoding in mmWave MIMO systems, successfully reducing pilot overhead but still being dependent on pre-training and centralized learning schemes.

Attention mechanisms were incorporated by An et al. [38] to enhance feature focus under noisy conditions, yet such networks carry substantial computational burdens. He and Yuan [39] proposed a cascaded DL model for intelligent metasurface-assisted MIMO, effectively handling passive element nonlinearities, but it is tailored for specialized infrastructure beyond typical WSN nodes.

Cherif et al. [40] employed autoencoders to mitigate nonlinear distortions in high-power amplifiers, though the approach requires precise parameter tuning and large-scale training datasets. Abdallah et al. [41] focused on frequency-selective DL-based estimation for hybrid MIMO systems, achieving strong performance in dynamic channels but facing challenges for real-time low-power sensor nodes.

Gao et al. [42] applied FFDNet for VLC-MIMO estimation, effectively modeling high-dimensional nonlinearities, yet the convolution-heavy design limits low-latency performance. Zha et al. [30] implemented adaptive predistortion using support vector regression for LED nonlinearity, which, while effective for VLC, remains inapplicable to RF domains.

A comprehensive survey by Saxena et al. [43] reviewed DL approaches in VLC systems, highlighting both their strengths and the persistent gap in real-time, dual-domain estimation capabilities. Dong et al. [44] explored CNN-based architectures that integrate spatial, frequency, and temporal correlations for mmWave massive MIMO estimation, approaching MMSE performance but with substantial memory and pilot overhead requirements.

Kalogerias and Petropulu [45] approached nonlinear channel estimation via Bayesian nonlinear filtering, introducing sequential gain map prediction for cooperative networks. Although offering accurate MMSE tracking, this method assumes ideal grid-based models. Both Maity et al. [46] and Rajput et al. [47] investigated decentralized DL-enhanced estimation frameworks for mmWave and MIMO WSNs under imperfect CSI, demonstrating robust hybrid transceiver designs but still requiring processing power beyond that of embedded WSN nodes.

In summary, while traditional ML, and DL approaches have provided valuable advances in nonlinear channel estimation, many fall short regarding hybrid adaptability, scalability, and computational efficiency, particularly within dynamic VLC-RF WSN environments. To address these multidimensional challenges, our proposed Hybrid VLC-RF channel estimation model introduces a lightweight, hierarchical, tree-based approach capable of real-time estimation, hybrid signal integration, and efficient computation, building directly upon the limitations identified across the current literature.

## 3. System Model

**Figure 2:**
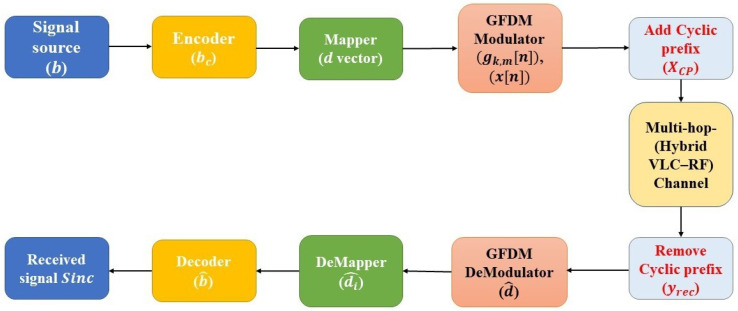
Block diagram of GFDM.

Throughout this model, we describe the end-to-end transmission and reception operations of a BPSK-based GFDM system deployed over a hybrid VLC-RF channel. The transmitter processes the binary data stream through channel coding using the BPSK modulation in GFDM, as well as cyclic prefixing. The signal then travels through a two-stage channel, where the first stage is a LoS-based VLC channel with AWGN, and the second stage is an RF Rayleigh fading channel. The receiver inverts the transmission chain using matched filtering, demapping, and decoding (see Figure 2).

In a GFDM environment, let the number of subcarriers be *K*, the number of subsymbols be *M*, and the total GFDM block length be N=KM. The total number of bits transmitted per block is $N_{b}$.

A. *Transmitter*

(1) Binary Source

(1) $$b=\left[b_{0},b_{1},\ldots ,b_{N_{b}-1}\right],\;b_{i}\in \left\{0,1\right\}.$$

This vector represents the raw binary information that the system aims to transmit. It is generated by the source and typically contains the payload data. Each element $b_{i}$ represents a single bit and forms the basis for modulation. The length $N_{b}$ depends on the block configuration and the channel coding parameters are used subsequently.

(2) Channel Encoder

(2) $$b_{c}=E\left(b\right),\;N_{c}=\frac{N_{b}}{r}.$$

To model the system’s bit-level processing behavior, the original bit vector b is mapped to a transformed bit sequence $b_{c}$ through the encoding function E(·). This captures any expansion or modification that may occur before modulation, such as bit padding. The transformation increases the bit vector size from $N_{b}$ to $N_{c}$, where r∈(0,1] represents a generic expansion factor. This step allows our system model to accommodate scenarios where additional bits are introduced intentionally or by system design before transmission.

(3) BPSK Mapping

(3) $$d_{i}=2b_{c,i}-1\;\Rightarrow \;d={\left[d_{0},d_{1},\ldots ,d_{N-1}\right]}^{⊤}.$$

Each encoded bit is mapped to a BPSK symbol: bit 0 becomes −1, and bit 1 becomes +1. This mapping results in a real-valued symbol vector $d\in {\left\{-1,+1\right\}}^{N}$. These symbols are later shaped and modulated by GFDM. BPSK was chosen for its robustness and simplicity, especially under low SNR and fading conditions, which, in turn, are a suitable choice for hybrid VLC-RF systems, where reliability is critical.

(4) GFDM Modulation

(4) $$g_{k,m}\left[n\right]=g\left[\left(n-mK\right)\;\mathrm{mod}\;N\right]\cdot e^{-j\frac{2\pi kn}{K}}.$$

The function $g_{k,m}\left[n\right]$ represents the time-frequency localized pulse shaping function applied to each data symbol $d_{k,m}$. It is a circularly time-shifted version of a filter g[n] modulated in frequency by the subcarrier index *k*. This ensures spectral containment while allowing overlap between subsymbols and subcarriers, which enhances bandwidth efficiency and time-frequency flexibility.

(5) $$x\left[n\right]=\sum_{k=0}^{K-1}\sum_{m=0}^{M-1}d_{k,m}\cdot g_{k,m}\left[n\right].$$

The total transmitted signal x[n] is formed by summing all shaped and modulated symbols over subcarriers and subsymbols, as defined by Equation (5). This results in a complex baseband signal of length *N*, which constitutes one GFDM block. By allowing overlapping in both the time and frequency domains, GFDM achieves improved spectral efficiency compared to OFDM, although this may introduce inter-symbol interference (ISI). Alternatively, the transmit signal can be expressed in a more compact and computationally efficient matrix form as x=A·d, where A is the modulation matrix composed of column vectors representing the filters $g_{k,m}\left[n\right]$, and d is the vector of data symbols. This matrix formulation not only simplifies the implementation, but also facilitates the analytical design of demodulators and equalizers.

(5) Cyclic Prefix Insertion

(6) $$x_{\mathrm{CP}}={\left[x\left[N-L_{\mathrm{CP}}\right],\ldots ,x\left[N-1\right],x\left[0\right],\ldots ,x\left[N-1\right]\right]}^{⊤}.$$

To eliminate inter-block interference and enable circular convolution at the receiver side, a cyclic prefix (CP) of length $L_{CP}$ was appended to the front of the signal. The CP duplicates the last $L_{CP}$ samples of the GFDM block, ensuring that the effects of channel delay spread do not corrupt the orthogonality of the modulation basis. This step is essential in systems with multipath propagation, especially the RF part of the hybrid channel.

B. *Hybrid VLC-RF Channel*

(1) VLC Channel (LoS + AWGN)

(7) $$y_{\mathrm{VLC}}=h_{\mathrm{vlc}}\cdot x_{\mathrm{CP}}+n_{\mathrm{vlc}},\;n_{\mathrm{vlc}}∼N\left(0,\sigma_{\mathrm{vlc}}^{2}I\right).$$

The VLC channel is modeled as a pure line-of-sight (LoS) channel with additive white Gaussian noise (AWGN). The channel gain $h_{\mathrm{LoS}}\in R_{+}$ represents the static optical gain, which depends on distance, LED angle, and receiver position. Since the environment is generally free of significant multipath scenarios, the VLC channel is deterministic and not faded. The noise $n_{\mathrm{vlc}}$ accounts for thermal, shot, and ambient light noise at the photodetector, and it is assumed to be uncorrelated Gaussian noise across time.

(2) RF Channel

(8) $$h_{\mathrm{RF}}∼\mathrm{CN}\left(0,\sigma_{h}^{2}\right),\;y=h_{\mathrm{RF}}\cdot y_{\mathrm{VLC}}+n_{\mathrm{rf}}\;\;,\;n_{\mathrm{rf}}∼\mathrm{CN}\left(0,\sigma_{\mathrm{rf}}^{2}I\right).$$

The signal from the VLC stage is transmitted through an RF channel characterized by Rayleigh fading, which captures the effects of three-path propagation in the RF domain. This is modeled using a channel impulse response $h_{\mathrm{RF}}$ as a complex Gaussian process. The delays in paths are defined as $\tau_{l}=\left[0,\;0.1,\;0.2\right]$ in (µs), and the corresponding power gains are $G_{l}=\left[0,-3,-6\right]$ dB. The operation (.) between $h_{\mathrm{RF}}$ and the VLC output introduces frequency selectivity and temporal dispersion, which is further impacted by the channel delay and power profiles. Additionally, additive white Gaussian noise (AWGN) $n_{\mathrm{rf}}$ is introduced due to the receiver circuitry and thermal noise in the RF stage. Eventually, the received signal from the hybrid VLC-RF channel (the signal passes sequentially through both the VLC and RF stages) can be expressed as

The VLC stage output is given by

(9) $$y_{\mathrm{VLC}}=h_{\mathrm{vlc}}\cdot x_{\mathrm{CP}}+n_{\mathrm{vlc}}.$$

Substituting $y_{\mathrm{VLC}}$ into the RF channel model yields

(10) $$y=h_{\mathrm{RF}}\cdot \left(h_{\mathrm{vlc}}\cdot x_{\mathrm{CP}}+n_{\mathrm{vlc}}\right)+n_{\mathrm{rf}},$$

which simplifies to

(11) $$\begin{array}{cc} y & =\left(h_{\mathrm{RF}}\cdot h_{\mathrm{vlc}}\right)\cdot x_{\mathrm{CP}}+h_{\mathrm{RF}}\cdot n_{\mathrm{vlc}}+n_{\mathrm{rf}}. \end{array}$$

Thus, the equivalent hybrid channel model can be compactly expressed as

(12) $$y=h_{\mathrm{eff}}\cdot x_{\mathrm{CP}}+n_{\mathrm{eff}},$$

where

(13) $$\begin{array}{cc} h_{\mathrm{eff}} & =h_{\mathrm{RF}}\cdot h_{\mathrm{vlc}}, \end{array}$$

(14) $$\begin{array}{cc} n_{\mathrm{eff}} & =h_{\mathrm{RF}}\cdot n_{\mathrm{vlc}}+n_{\mathrm{rf}}. \end{array}$$

Here, $h_{\mathrm{vlc}}$ and $h_{\mathrm{RF}}$ represent the VLC and RF channel coefficients, respectively. The effective channel $h_{\mathrm{eff}}$ is given by the product of the two individual channels, corresponding to a sequential transmission through both. The total noise $n_{\mathrm{eff}}$ consists of the VLC noise term $n_{t,\mathrm{vlc}}$, which is scaled by the RF channel, and the additive noise $n_{t,\mathrm{rf}}$ from the RF stage.

To estimate the effective hybrid channel $h_{\mathrm{eff}}=h_{\mathrm{vlc}}\cdot h_{\mathrm{RF}}$, we generated supervised learning pairs by simulating the hybrid channel’s output y for known transmitted signals $x_{\mathrm{CP}}$ under varying SNR conditions. The known effective channel coefficients from te simulation serve as ground-truth labels. Our algorithms are trained to learn the nonlinear mapping from the received signal y to the estimated channel response ${\hat{h}}_{\mathrm{eff}}$. This data-driven approach enables robust estimation, even under nonlinear and noisy hybrid VLC-RF conditions.

C. *Receiver*

(1) CP Removal

The receiver removes the cyclic prefix from the received signal.

(15) $$y_{\mathrm{rec}}=y\left[L_{CP}:L_{CP}+N-1\right].$$

This step restores the original structure of the GFDM block and ensures that subsequent demodulation can assume circular convolution. This is necessary for matched filtering using the GFDM modulation matrix, which relies on circular signal alignment.

(2) GFDM Demodulation

(16) $$\hat{d}=A^{H}\cdot y_{\mathrm{rec}}.$$

Demodulation is performed using the Hermitian transpose of the modulation matrix, implementing a matched filter receiver. This maps the received signal onto the known transmit basis functions and suppresses uncorrelated noise. While the process seems simple, the matched filter may allow residual self-interference in GFDM systems due to the non-orthogonality of the modulation basis.

(3) BPSK Demapping

(17) $${\hat{b}}_{i}=\left\{\begin{array}{cc} 0, & ℜ({\hat{d}}_{i})<0\\ 1, & ℜ({\hat{d}}_{i})\geq 0. \end{array}\right.$$

Each demodulated symbol is thresholded to its nearest BPSK constellation point. Since BPSK symbols are real-valued, the decision boundary is at zero. This slicing operation reverts the continuous-valued detector outputs to binary decisions, providing an estimated bitstream for decoding.

(4) Channel Decoding

(18) $$\hat{b}=E^{-1}\left({\hat{b}}_{c}\right).$$

The decoder inverts the FEC encoding operation using the added redundancy to detect and correct bit errors. This step is crucial in recovering the original message when operating under noisy or fading conditions. The quality of decoding depends on the SNR (whose values range from −5 to 30 dB) and the strength of the channel coding process (see Table 1).

## 4. Model Configuration and Training Parameters

This section outlines the numerical configuration of all AI-based models employed for channel estimation in hybrid VLC-RF GFDM systems. The chosen parameter values were carefully selected to balance model complexity, accuracy, and computational efficiency, ensuring robust performance across varying SNR conditions. The tree model was configured with 100 decision trees, each with a maximum of 160 splits to fully leverage the 160 input features, and 160 independent regressors were trained to estimate each output component. The random forest model followed a similar structure, using 100 trees with 13 predictors randomly selected at each split to encourage diversity among trees and a minimum leaf size of 5 to optimize generalization. For support vector machine (SVM) models, a linear kernel was used in combination with standardized input features to ensure efficient training and reliable prediction. The SVM configuration included a BoxConstraint of 1 and an Epsilon value of 0.1, which control the margin softness and the regression sensitivity zone, respectively. The linear regression models were implemented using ordinary least squares with a linear polynomial degree, offering fast training and interpretability. All models were trained using a dataset comprising 18,000 samples and 160 features, generated across 36 distinct SNR levels with 500 signal realizations per level. The dataset was partitioned into 70% for training and 30% for testing, and the numerical parameters listed contributed directly to the strong generalization and predictive capabilities observed in the AI-based estimators (see Table 2).

The first approach utilizes a tree model based on bagging (bootstrap aggregation). This method was chosen for its ability to reduce variance and capture nonlinear patterns through multiple decision trees trained independently for each output. The Algorithm 1 outlines the full training and evaluation process for the tree-based model.

The second method applies a random forest regressor, which extends the tree approach by introducing feature randomness at each split. This helps prevent overfitting and improves generalization. The Algorithm 2 below describes the training and evaluation pipeline for the random forest model, including parameter settings, such as the number of trees and predictors per split.

The third technique is based on support vector machines using a linear kernel. SVMs are effective for high-dimensional data and provide stable, interpretable decision boundaries. The Algorithm 3 details the steps for training and testing individual SVM models for each output bit, including input standardization and prediction thresholding.

The final model uses ordinary least squares (OLS) linear regression. While simpler than tree- and kernel-based methods, linear regression provides a useful performance baseline. The Algorithm 4 below summarizes the training of 160 independent linear models and the subsequent evaluation using classification metrics.
**Algorithm 1** Tree decision-based training and evaluation (70/30 split). **Inputs:**       Dataset: dataset[1..18,000][1..160]       Labels: label[1..18,000] (Integer) **Outputs:**       Trained Model: TrModel       Test Accuracy, Precision, Recall, F1-score  1:Define train_set[1..12,600][1..160], test_set[1..5400][1..160]  2:Define train_label[1..12,600], test_label[1..5400]  3:Define TrMod, prediction[1..5400], correct←0  4:**// Step 1: Split dataset into 70% training and 30% testing**  5:**for** i←1 **to** 12,600 **do**  6:    **for** j←1 **to** 160 **do**  7:        train_set[i][j]←dataset[i][j]  8:    **end for**  9:    train_label[i]←label[i]10:**end for**11:**for** i←12,601 **to** 18,000 **do**12:    **for** j←1 **to** 160 **do**13:        test_set[i−12,600][j]←dataset[i][j]14:    **end for**15:    test_label[i−12,600]←label[i]16:**end for**17:**// Step 2: Train Tree-Based Model**18:TrMod←fitctree(train_set, train_label)19:**// Step 3: Test the Model**20:**for** i←1 **to** 5400 **do**21:    prediction[i]←Predict(TrMod,test_set[i])22:    **if** prediction[i]=test_label[i] **then**23:        correct←correct+124:    **end if**25:**end for**26:**// Step 4: Calculate Metrics**27:$\mathrm{accuracy}\leftarrow \left(\frac{\mathrm{TP}+\mathrm{TN}}{\mathrm{TP}+\mathrm{FP}+\mathrm{TN}+\mathrm{FN}}\right)\times 100$28:$\mathrm{precision}\leftarrow \frac{\mathrm{TP}}{\mathrm{TP}+\mathrm{FP}}$29:$\mathrm{recall}\leftarrow \frac{\mathrm{TP}}{\mathrm{TP}+\mathrm{FN}}$30:f1_score $\leftarrow 2\times \frac{\mathrm{precision}\times \mathrm{recall}}{\mathrm{precision}+\mathrm{recall}}$

**Algorithm 2** Random forest training and evaluation (70/30 split).
 **Inputs:**       Dataset: dataset[1..18,000][1..160]       Target: target[1..18,000][1..160] **Outputs:**       Trained Model: RFModel[1..160]       Test Accuracy, Precision, Recall, F1-score
  1:
**// Step 1: Split Dataset into 70% Training and 30% Testing**
  2:Define train and test sets for inputs and targets  3:
**// Step 2: Train Random Forest Models**
  4:**for** k←1 to 160 **do**  5:    RFModel[k]←TreeBagger(100,train_set,train_label[:,k],
           ‘NumPredictorsToSample’ = 13, ‘MinLeafSize’ = 5)
  6:
**end for**
  7:
**// Step 3: Test the Model**
  8:**for** i←1 to 5400 **do**  9:    **for** k←1 to 160 **do**10:        prediction[i][k]←predict(RFModel[k],test_set[i])11:    **end for**12:
**end for**
13:
**// Step 4: Calculate Metrics**
14:

$\mathrm{accuracy}\leftarrow \left(\frac{\mathrm{TP}+\mathrm{TN}}{\mathrm{TP}+\mathrm{FP}+\mathrm{TN}+\mathrm{FN}}\right)\times 100$

15:

$\mathrm{precision}\leftarrow \frac{\mathrm{TP}}{\mathrm{TP}+\mathrm{FP}}$

16:

$\mathrm{recall}\leftarrow \frac{\mathrm{TP}}{\mathrm{TP}+\mathrm{FN}}$

17:f1_score $\leftarrow 2\times \frac{\mathrm{precision}\times \mathrm{recall}}{\mathrm{precision}+\mathrm{recall}}$


**Algorithm 3** Support vector machine training and evaluation (70/30 split).
 **Inputs:**       Dataset: dataset[1..18,000][1..160]       Target: target[1..18,000][1..160] **Outputs:**       Trained Model: SVMModel[1..160]       BER, Accuracy, Precision, Recall, F1-score
  1:
**// Step 1: Normalize and Split Dataset**
  2:Standardize dataset and split into training and testing sets  3:
**// Step 2: Train SVM Models**
  4:**for** k←1 to 160 **do**  5:    SVMModel[k]←fitrsvm(train_set,train_label[:,k],
           ‘KernelFunction’ = ‘linear’, ‘Standardize’ = true)
  6:
**end for**
  7:
**// Step 3: Test the Model**
  8:**for** i←1 to 5400 **do**  9:    **for** k←1 to 160 **do**10:        prediction[i][k]←predict(SVMModel[k],test_set[i])11:    **end for**12:
**end for**
13:
**// Step 4: Calculate Metrics**
14:

$\mathrm{accuracy}\leftarrow \left(\frac{\mathrm{TP}+\mathrm{TN}}{\mathrm{TP}+\mathrm{FP}+\mathrm{TN}+\mathrm{FN}}\right)\times 100$

15:

$\mathrm{precision}\leftarrow \frac{\mathrm{TP}}{\mathrm{TP}+\mathrm{FP}}$

16:

$\mathrm{recall}\leftarrow \frac{\mathrm{TP}}{\mathrm{TP}+\mathrm{FN}}$

17:f1_score $\leftarrow 2\times \frac{\mathrm{precision}\times \mathrm{recall}}{\mathrm{precision}+\mathrm{recall}}$


**Algorithm 4** Linear Regression Training and Evaluation (70/30 Split)
 **Inputs:**       Dataset: dataset[1..18,000][1..160]       Target: target[1..18,000][1..160] **Outputs:**       Trained Model: LRModel[1..160]       BER, Accuracy, Precision, Recall, F1-score
  1:
**// Step 1: Split Dataset**
  2:Partition dataset into 70% training and 30% testing subsets  3:
**// Step 2: Train Linear Models**
  4:**for** k←1 to 160 **do**  5:    LRModel[k]←fitlm(train_set,train_label[:,k])  6:
**end for**
  7:
**// Step 3: Test the Model**
  8:**for** i←1 to 5400 **do**  9:    **for** k←1 to 160 **do**10:        prediction[i][k]←predict(LRModel[k],test_set[i])11:    **end for**12:
**end for**
13:
**// Step 4: Calculate Metrics**
14:

$\mathrm{accuracy}\leftarrow \left(\frac{\mathrm{TP}+\mathrm{TN}}{\mathrm{TP}+\mathrm{FP}+\mathrm{TN}+\mathrm{FN}}\right)\times 100$

15:

$\mathrm{precision}\leftarrow \frac{\mathrm{TP}}{\mathrm{TP}+\mathrm{FP}}$

16:

$\mathrm{recall}\leftarrow \frac{\mathrm{TP}}{\mathrm{TP}+\mathrm{FN}}$

17:f1_score $\leftarrow 2\times \frac{\mathrm{precision}\times \mathrm{recall}}{\mathrm{precision}+\mathrm{recall}}$


## 5. Performance Metrics

To evaluate the performance of the proposed model, we employed several standard classification metrics: accuracy, precision, recall, F1-score, and inference time. These metrics provide a comprehensive understanding of the model’s effectiveness and efficiency.

### 5.1. Accuracy

Accuracy measures the proportion of correctly predicted bits (both (−1 s) and (1 s) in BPSK modulation) out of the total number of bits across all test data samples. It is defined as

(19) $$\mathrm{Accuracy}=\frac{TP+TN}{TP+TN+FP+FN},$$

where TP is True Positive and means a bit is actually 1, and the model correctly predicted it as 1; TN is True Negative and it represents a bit is actually −1, and the model correctly predicted it as −1; FP is False Positive and means a bit is actually −1, but the model incorrectly predicted it as 1; and FN is False Negative which means a bit is actually 1, but the model incorrectly predicted it as −1.

### 5.2. Precision

Precision measures how many of the bits predicted as 1 were, in actuality, 1, in other words, it is the proportion of true positives among all positives predicted by the model, and it is expressed as

(20) $$\mathrm{Precision}=\frac{TP}{TP+FP}.$$

### 5.3. Recall

Recall (also known as sensitivity or true positive rate) measures how many of the actual 1 bits were correctly predicted by the model, meaning the proportion of true positives among all actual positives.

(21) $$\mathrm{Recall}=\frac{TP}{TP+FN}.$$

### 5.4. F1-Score

F1-score represents the harmonic mean of precision and recall, offering a single measure that balances both false positives and false negatives in bit-level estimation, and it is expressed as

(22) $$F1\text{-}\mathrm{Score}=2\cdot \frac{\mathrm{Precision}\cdot \mathrm{Recall}}{\mathrm{Precision}+\mathrm{Recall}}.$$

### 5.5. Inference Time

Inference time refers to the total time required by the model (after training) to estimate all bits across the 5400 testing samples, each with 160 bits (for a total of 864,000 predictions). It is defined as

(23) $$\mathrm{Inference}\;\mathrm{Timetotal}=\sum {i=1}^{N}t_{i},$$

where

*N* is the total number of test samples;

$t_{i}$ is the inference time for the *i*th test sample.

### 5.6. Bit Error Rate (BER)

The Bit Error Rate (BER) indicates the ratio of incorrectly predicted bits (errors) to the total number of transmitted bits across the dataset. It is given by

(24) $$\mathrm{BER}=\frac{\mathrm{Number}\;\mathrm{of}\;\mathrm{Bit}\;\mathrm{Errors}}{\mathrm{Total}\;\mathrm{Number}\;\mathrm{of}\;\mathrm{Transmitted}\;\mathrm{Bits}}.$$

## 6. Simulation and Results

To evaluate the performance of the proposed tree decision-based model for hybrid VLC-RF channel estimation in GFDM wireless sensor networks, we conducted simulations across a wide SNR range from −5 dB to 30 dB. The tree model was compared with five other methods: Minimum Mean Square Error (MMSE), Linear MMSE (LMMSE), support vector machine (SVM), random forest (RF), and linear regression. This comparison includes both traditional statistical estimators and machine learning models to ensure comprehensive benchmarking.

**Figure 3:**
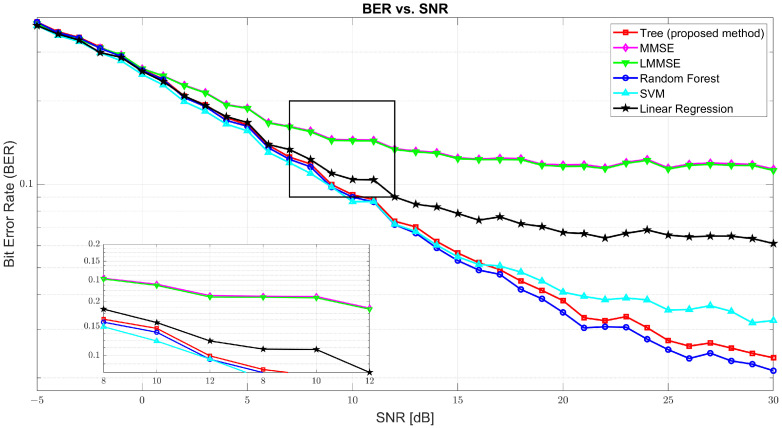
The Bit Error Rate (BER) vs. SNR for tree, MMSE, LMMSE, SVM, RF, and linear regression.

Figure 3 illustrates the BER performance. At SNR = 0 dB, BER was found to be high across all models due to significant noise, with tree at 0.2591, MMSE at 0.2615, and RF slightly better at 0.2577. As the SNR increased, BER sharply declined. At 10 dB, the tree model improved drastically to 0.0917, while MMSE and LMMSE remained relatively high at 0.1448 and 0.1438, respectively. RF and SVM performed closely to tree with 0.0899 and 0.0866, respectively. At 30 dB, tree achieved an excellent BER of 0.0237, only slightly behind RF (0.0212) and SVM (0.0323), and far ahead of MMSE (0.1135) and LMMSE (0.1122), confirming tree’s robustness in high SNR environments.

The inference time presented in Table 3 refers to the total duration required by each model to complete the prediction process on the entire test set. Specifically, with a dataset consisting of 18,000 samples and a 70/30 train–test split, each model performed inference on 5400 test samples. Given that each sample had 160 output features, this resulted in a total of 864,000 individual predictions per model. Inference time strictly measures how quickly a trained model can generate these predictions during the deployment phase, excluding any training or preprocessing time. This inference time is crucial in evaluating the practicality of different models, particularly for real-time and latency-sensitive applications where fast and efficient prediction is essential.

While random forest achieved slightly superior BER performance across most SNR levels, the proposed tree model offers a compelling trade-off between accuracy and inference efficiency. As shown in Figure 3, tree achieved BER results close to the best-performing models: at 10 dB, it recorded a BER of 0.0956 compared to 0.0840 for random forest; and, at 30 dB, it reached 0.0216, only marginally behind RF’s 0.0146. However, the inference time, as detailed in Table 3, highlighted a crucial advantage of the tree model. On a test set of 5400 samples with 864,000 total predictions, the tree model completed inference in 45.53 s, significantly faster than random forest (140.09 s) and SVM (189.35 s). This considerable speed-up that is 3 times faster than RF and 4 times faster than SVM makes the tree model more suitable for real-time and latency-sensitive applications, where inference time is often as critical as prediction accuracy. Thus, despite not having the absolute lowest BER, the tree model attains a practical balance between robust accuracy and efficient inference, which makes it a strong candidate for deployment in real-world systems.

**Figure 4:**
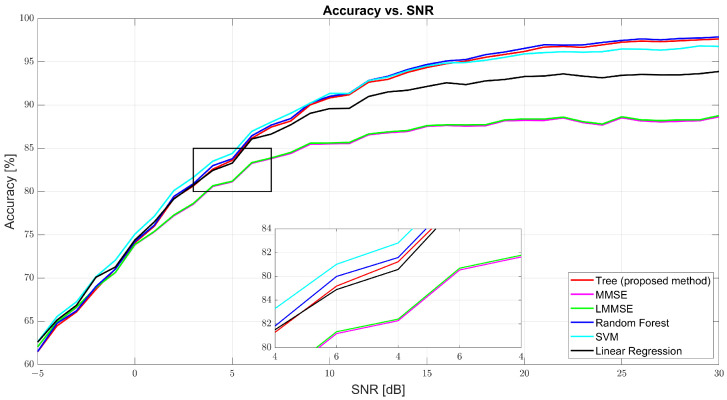
Regression accuracy vs. SNR for tree, MMSE, LMMSE, SVM, RF, and linear regression.

As shown in Figure 4, regression accuracy improves with SNR. At 0 dB, the accuracy was modest: tree (74.09%), MMSE (73.85%), and RF (74.23%). Despite low values, the ML models already began outperforming the traditional estimators. At 10 dB, tree surged to 90.83%, while MMSE and LMMSE lagged at 85.52% and 85.62%. RF led with 91.01%, and SVM followed closely (91.34%). At 30 dB, tree achieved 97.63%, slightly behind RF (97.88%) and well above MMSE (88.65%) and LMMSE (88.78%). These results highlight tree’s scalability and data adaptability as the SNR improves.

**Figure 5:**
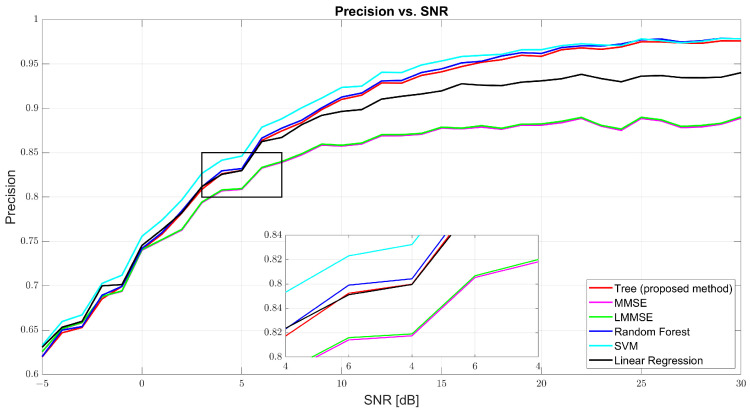
Precision vs. SNR for tree, MMSE, LMMSE, SVM, RF, and linear regression.

**Figure 6:**
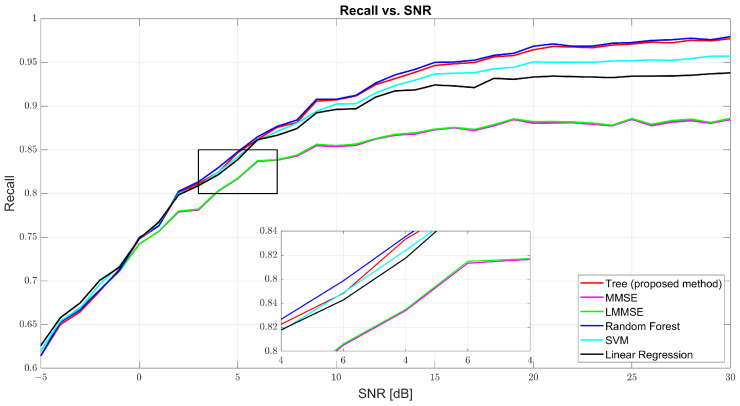
Recall vs. SNR for tree, MMSE, LMMSE, SVM, RF, and linear regression.

Figure 5 and Figure 6 show that both precision and recall grew significantly with the SNR. At 0 dB, tree’s precision was 61.98% and its recall was 61.41%, while RF and SVM were slightly better (62–63%). At 10 dB, tree’s precision was 90.57% and its recall was 90.40%, just behind RF (91.27% and 91.01%, respectively) and SVM (91.09% and 90.91%, respectively). At 30 dB, tree achieved 97.89% precision and 97.83% recall, demonstrating strong balance. MMSE and LMMSE plateaued around 89%, showing limited adaptability, even at high SNR.

**Figure 7:**
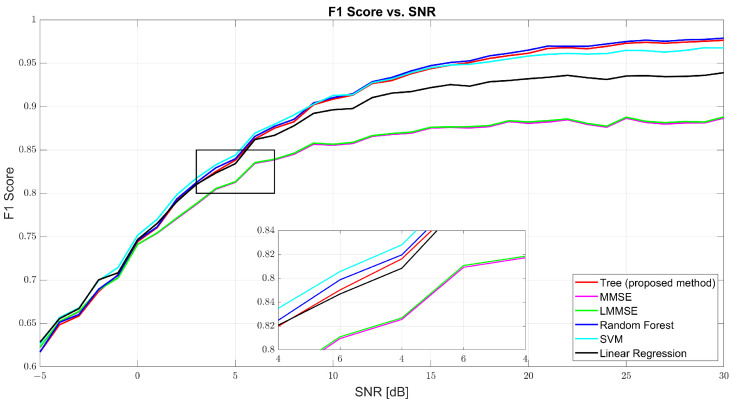
F1-score vs. SNR for tree, MMSE, LMMSE, SVM, RF, and linear regression.

Figure 7 further confirms the tree model’s well-rounded performance through the F1-score curve. At 0 dB, tree scored 61.69%, with RF and SVM close behind. At 10 dB, tree reached 90.48% compared to MMSE’s 86% and RF’s 91.13%. At 30 dB, the tree model hit 97.86%, closely trailing RF (98.22%) and outperforming SVM (97.31%) and linear regression (95.94%). The consistent rise of the tree curve indicates its ability to maintain both high precision and high recall as channel conditions improve.

**Figure 8:**
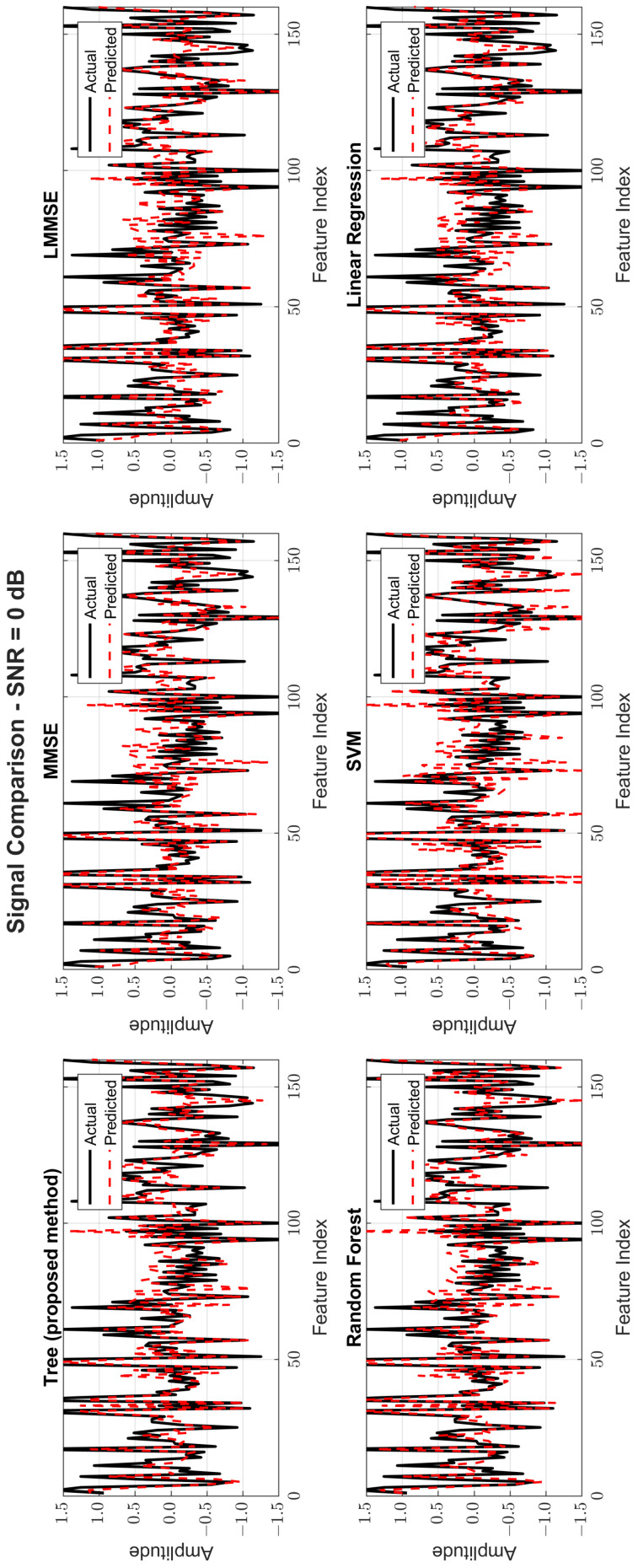
Original and estimated signals at SNR = 0 dB for all models.

**Figure 9:**
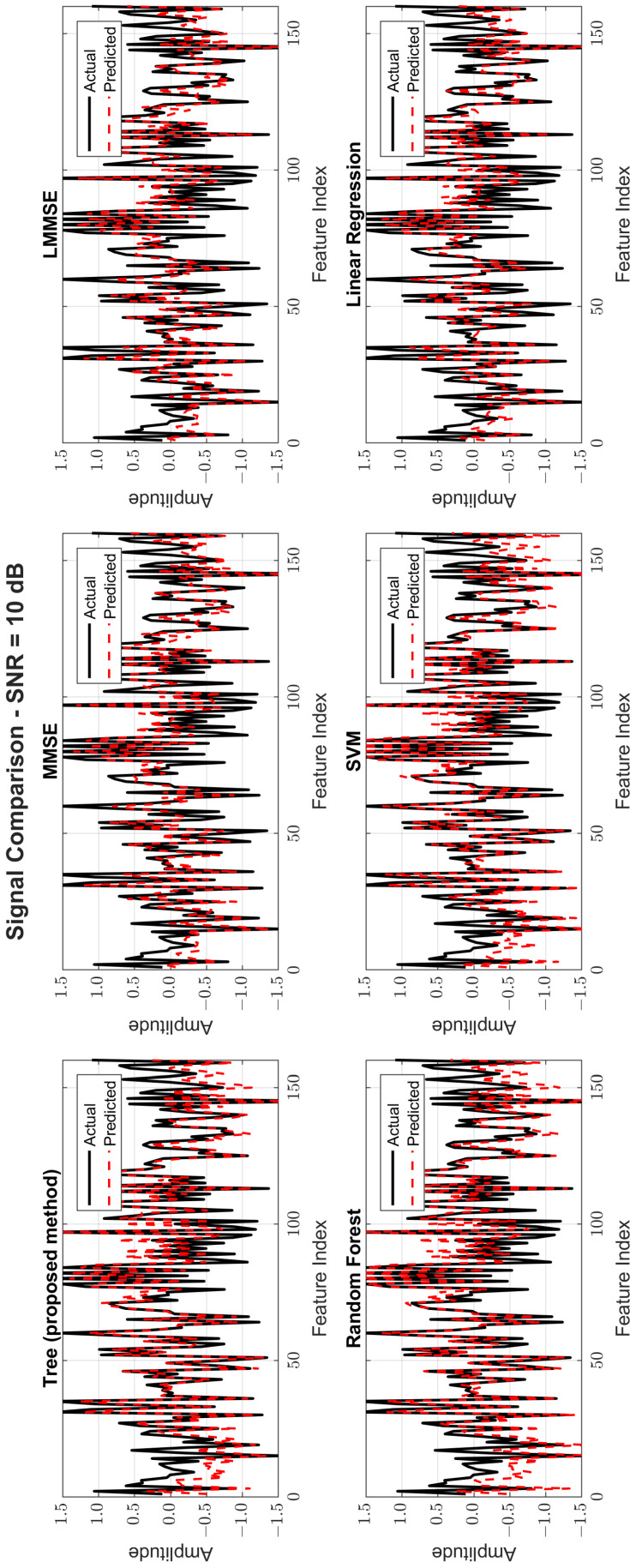
Original and estimated signals at SNR = 10 dB for all models.

**Figure 10:**
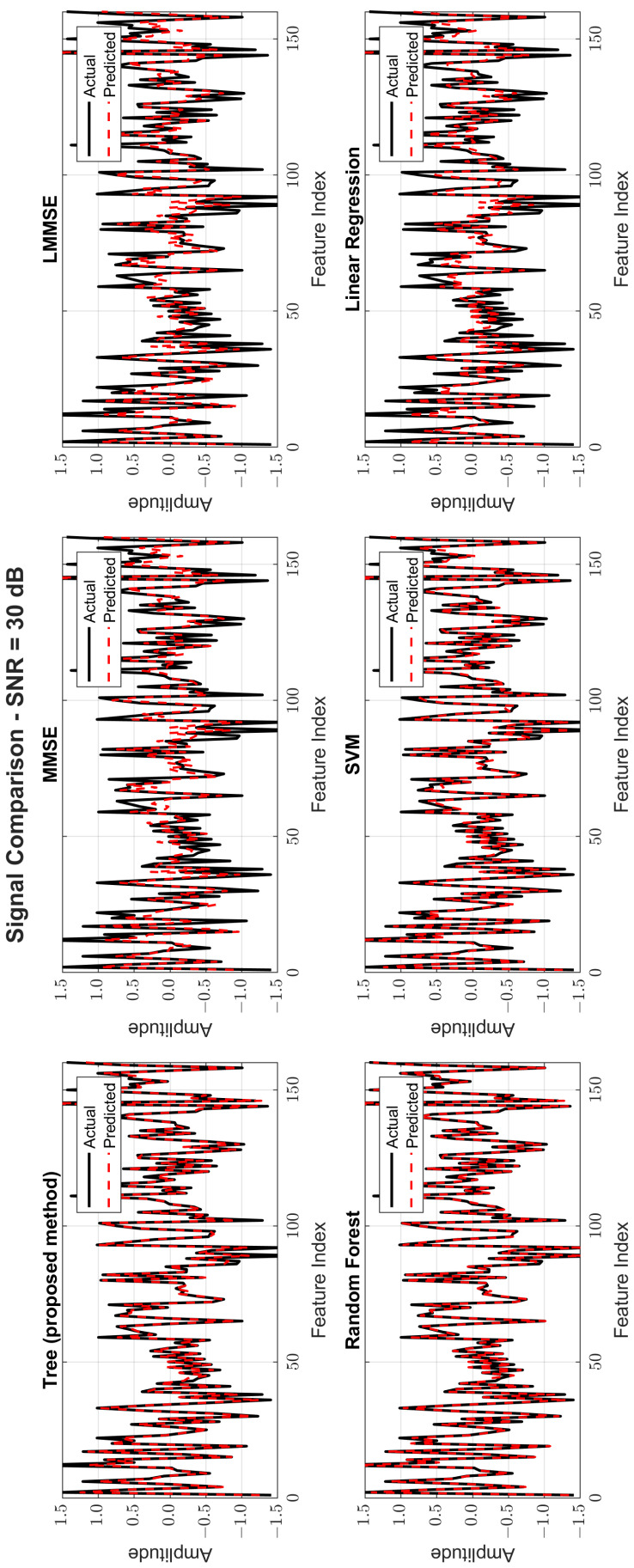
Original and estimated signals at SNR = 30 dB for all models.

Figure 8, Figure 9 and Figure 10 provide signal estimation plots. At 0 dB, the tree model retained more signal shape compared to MMSE, which displayed smoothing and attenuation. At 10 dB, tree better approximated peaks and valleys, with RF and SVM also showing improvement. At 30 dB, the tree model’s estimated waveform was nearly indistinguishable from the original signal, demonstrating excellent structural fidelity, and it was similar to RF and superior to the traditional estimators.

**Figure 11:**
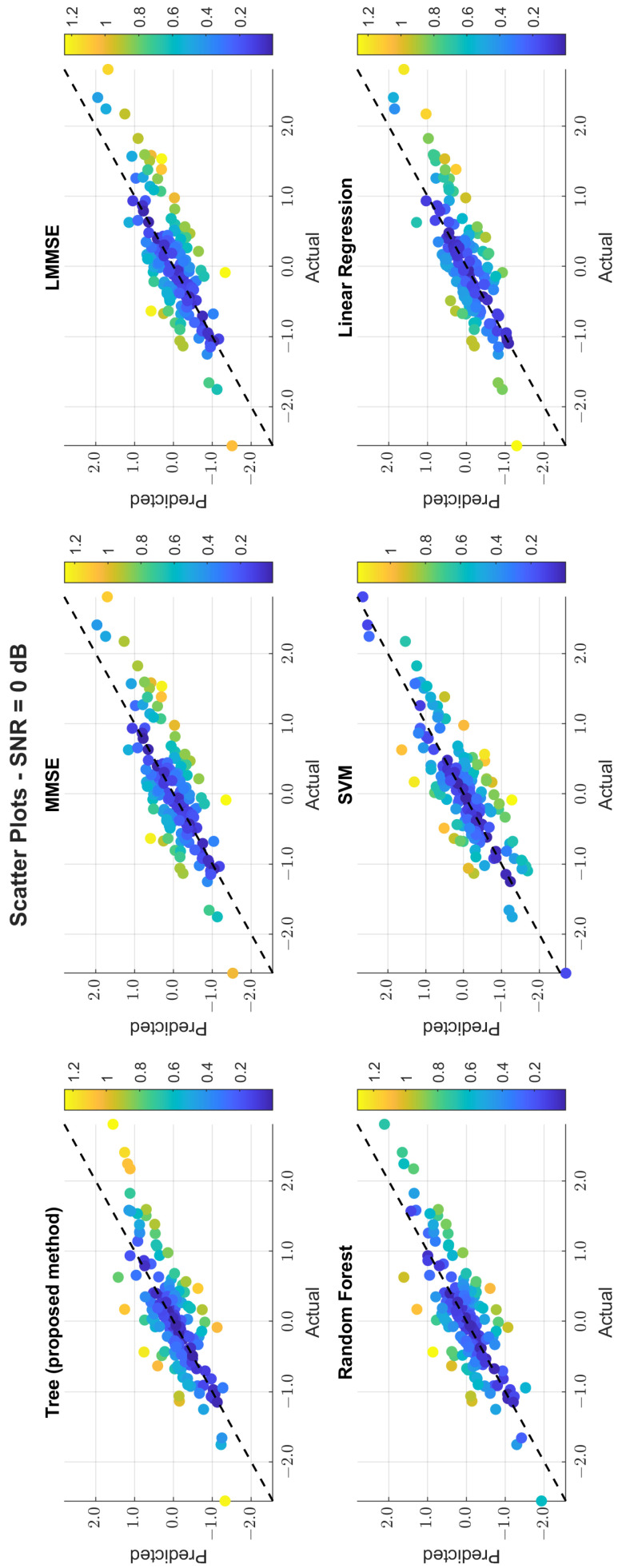
Scatter plot of the predicted vs. actual values at SNR = 0 dB.

**Figure 12:**
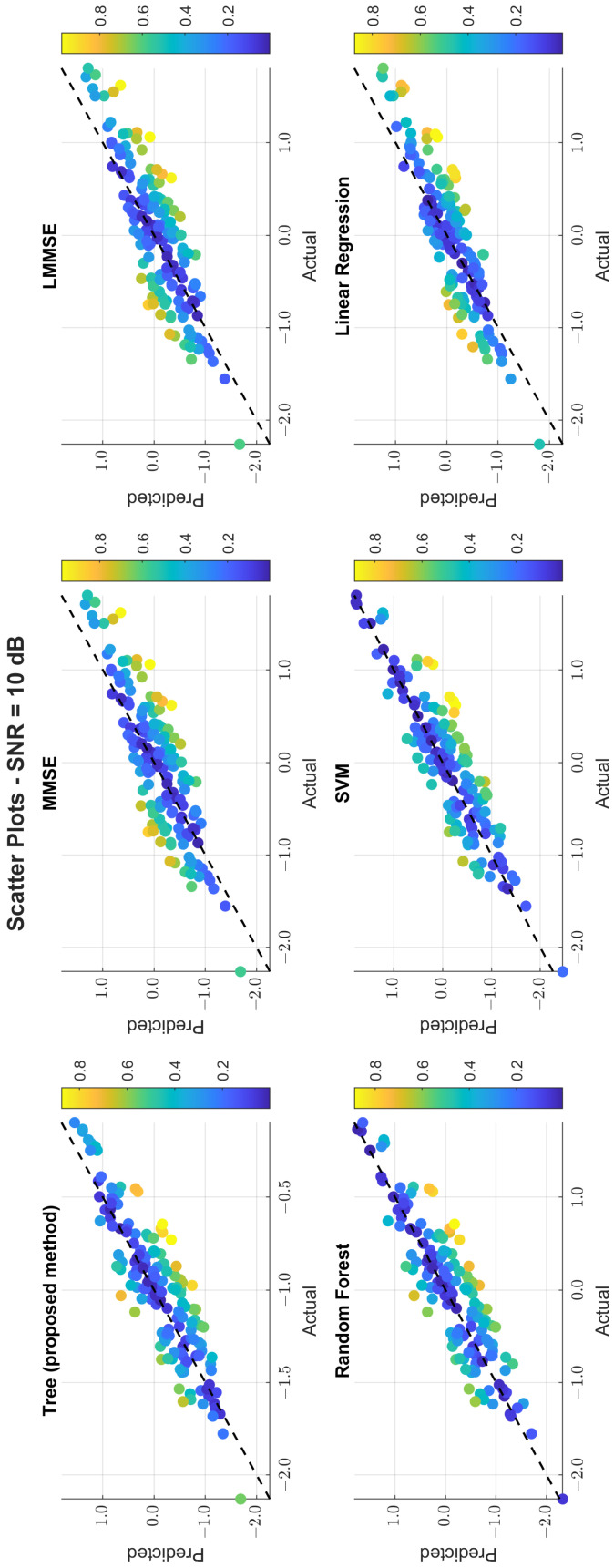
Scatter plot of the predicted vs. actual values at SNR = 10 dB.

**Figure 13:**
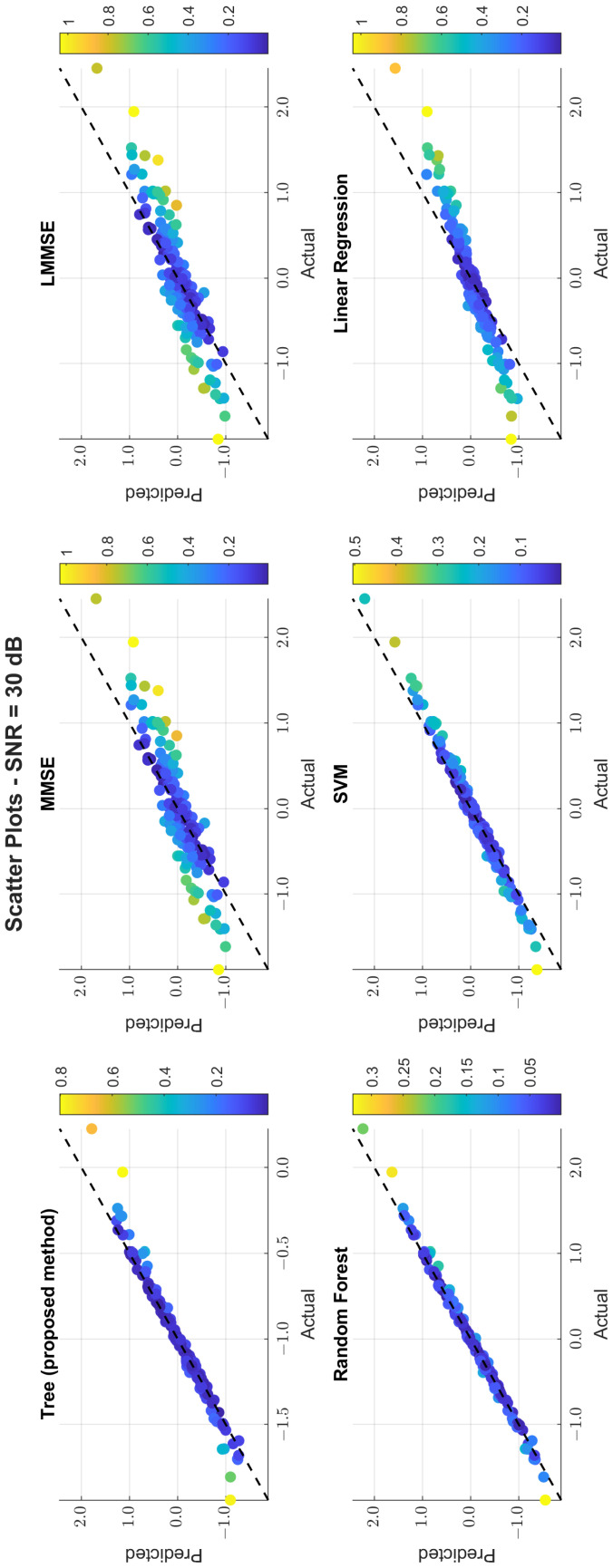
Scatter plot of the predicted vs. actual values at SNR = 30 dB.

The scatter plots in Figure 11, Figure 12 and Figure 13 illustrate the prediction error. At 0 dB, tree showed moderate spread, while MMSE/LMMSE exhibited significant variance. At 10 dB, tree points began clustering along the ideal diagonal, reflecting stronger correlation. At 30 dB, tree and RF produced tight, diagonal-aligned clouds, indicating minimal bias and high regression precision. MMSE still showed offset and greater variance.

Beyond accuracy and error metrics, inference time is a key consideration for real-time channel estimation in wireless sensor networks. Table 3 presents the average inference time for each model. As expected, classical estimators, such as MMSE (0.007374 s) and LMMSE (0.009032 s), were the fastest due to their low computational complexity. Linear regression also performed reasonably well with an inference time of 1.181935 s, although its estimation accuracy was limited.

The proposed tree model required an average inference time of 45.531617 s. While this was significantly higher than that of MMSE and linear regression, it was still substantially lower than random forest (140.094001 s) and SVM (189.347879 s), which offer only marginal performance improvements in some scenarios. The tree model thus achieved a practical balance—delivering high estimation accuracy and low BER across all SNR levels while maintaining moderate computational demand. This makes it particularly well suited for deployment in real-world systems that require a compromise between performance and latency.

In contrast, although the random forest and SVM models provided strong accuracy, their high inference times may limit applicability in time-sensitive or energy-constrained WSN environments. The tree model, therefore, offers an attractive middle ground: it outperforms traditional methods and avoids the excessive computational load associated with more complex ML ensembles (see Table 4).

## 7. Conclusions

This paper introduced a tree decision-based regression model for channel estimation in hybrid VLC-RF generalized frequency division multiplexing (GFDM) wireless sensor networks. Through comprehensive simulations across a broad range of SNR values from −5 dB to 30 dB, the proposed model was benchmarked against five well-established techniques: Minimum Mean Square Error (MMSE), linear MMSE (LMMSE), support vector machine (SVM), random forest (RF), and linear regression.

The tree model consistently achieved very good performance across the key evaluation metrics, particularly in the mid-to-high SNR region, where most practical wireless systems operate. At SNR = 10 dB, the tree model achieved an accuracy of **90.83%** with a BER of **0.0917**. In contrast, MMSE and LMMSE lagged at around **85.5%** accuracy, while random forest slightly led with **91.01%** and SVM achieved **91.34%**. At this level, the tree model also reduced the Bit Error Rate (BER) to **0.0917**, outperforming MMSE (**0.1448**) and LMMSE (**0.1438**), as well as closely tracking random forest (**0.0899**) and SVM (**0.0866**).

At SNR = 30 dB, the tree-based model further improved, achieving **97.63%** accuracy with a BER of **0.0237**. The BER of the proposed model dropped to a value nearly **five times lower** than MMSE (**0.1135**) and closely matched random forest (**0.0212**) and SVM (**0.0323**). These results validate the tree model’s ability to maintain robustness not only in ideal high-SNR conditions, but also in the more critical and realistic mid-SNR regime, where conventional estimators begin to saturate in performance.

Moreover, the tree-based model achieved this balance with a moderate inference time of **45.53 s**, which was significantly faster than random forest (**140.09 s**) and SVM (**189.35 s**), as well as only moderately higher than linear regression (**1.1819 s**). While the MMSE **(0.0073 s)** and LMMSE **(0.0090 s)** models were faster, they lacked the accuracy and BER required in dynamic environments. Thus, the tree model offers an effective trade-off between accuracy and computational efficiency, making it highly suitable for time-sensitive WSN deployments.

## Figures and Tables

**Table 1:** Parameter descriptions for the GFDM-based hybrid VLC-RF system.

Parameter	Value/Description
**GFDM Parameters**	
Number of subcarriers (*K*)	16
Number of time slots (*M*)	10
Total GFDM symbol length (N=K×M)	160
Modulation scheme	BPSK
Roll-off factor (α)	0.25
Cyclic prefix length	16 samples
**VLC Channel Parameters**	
Channel type	AWGN
Noise type	Additive white Gaussian noise
SNR range	−5 dB to 30 dB
**RF Channel Parameters**	
Channel model	Rayleigh fading
Number of paths (*L*)	3
Path delays ($\tau_{l}$)	[0,1,2]μs
Path gains ($G_{l}$)	[0,−3,−6] dB
**Simulation Parameters**	
SNR range tested	−5 to 30 dB
Signals per SNR	500
Monte Carlo runs	50
Dataset structure	Input, target, and label per sample

**Table 2:** The numeric parameters used in the AI-based channel estimation models.

Model	Parameter	Value
Tree	Number of Trees	100
	Max Number of Splits per Tree	160
	Number of Features per Sample	160
	Number of Samples	18,000
	SNR values	36
	Signals per SNR	500
	Train/Test Split	70% Train/30% Test
Random Forest	Number of Trees	100
	Predictors per Split	13 (approx. $\sqrt{160}$)
	Minimum Leaf Size	5
SVM	Kernel Type	Linear
	Standardization	Enabled
	BoxConstraint	1
	Epsilon	0.1
Linear Regression	Polynomial Degree	1
Hardware	Device Model	Dell Vostro 15 3510
	Processor	11th Gen Intel(R) Core(TM) i7-1165G7 @ 2.80GHz
	Installed RAM	16.0 GB (3200 MHz)
	Graphics Card	2 GB (Multiple GPUs installed)
	Storage	1.38 TB (840 GB used)
	Operating System	Windows 64-bit (x64-based processor)
	Software	MATLAB 2021a

**Table 3:** The average inference time of each estimator.

Model	Inference Time (s)
MMSE	0.007374
LMMSE	0.009032
Linear Regression	1.181935
Random Forest	140.094001
SVM	189.347879
Tree (Proposed)	45.531617

**Table 4:** The BER and accuracy of all the models from a SNR of −5 to 30.

SNR	Tree (Our)		MMSE		LMMSE		RandomForest		SVM		LinearRegression	
	BER	Acc	BER	Acc	BER	Acc	BER	Acc	BER	Acc	BER	Acc
−5	0.3853	0.6147	0.3798	0.6202	0.3795	0.6205	0.3851	0.6149	0.3739	0.6261	0.3742	0.6258
−4	0.3552	0.6448	0.3511	0.6489	0.3509	0.6491	0.3523	0.6477	0.3445	0.6555	0.3485	0.6515
−3	0.3392	0.6608	0.3341	0.6659	0.3337	0.6663	0.3379	0.6621	0.3274	0.6726	0.3315	0.6685
−2	0.3130	0.6870	0.3110	0.6890	0.3108	0.6892	0.3097	0.6903	0.2984	0.7016	0.2990	0.7010
−1	0.2893	0.7107	0.2936	0.7065	0.2934	0.7066	0.2896	0.7104	0.2792	0.7208	0.2871	0.7128
0	0.2591	0.7409	0.2615	0.7385	0.2612	0.7388	0.2577	0.7423	0.2492	0.7508	0.2559	0.7441
1	0.2399	0.7601	0.2464	0.7536	0.2460	0.7540	0.2388	0.7612	0.2284	0.7716	0.2350	0.7650
2	0.2080	0.7920	0.2280	0.7720	0.2271	0.7729	0.2056	0.7944	0.1989	0.8011	0.2085	0.7915
3	0.1935	0.8065	0.2146	0.7854	0.2137	0.7863	0.1909	0.8091	0.1836	0.8164	0.1924	0.8076
4	0.1741	0.8259	0.1941	0.8059	0.1933	0.8067	0.1700	0.8300	0.1649	0.8351	0.1755	0.8245
5	0.1639	0.8361	0.1887	0.8113	0.1880	0.8120	0.1621	0.8379	0.1560	0.8440	0.1671	0.8329
6	0.1384	0.8616	0.1673	0.8327	0.1665	0.8335	0.1355	0.8645	0.1304	0.8696	0.1392	0.8608
7	0.1254	0.8746	0.1618	0.8382	0.1611	0.8389	0.1231	0.8769	0.1197	0.8803	0.1335	0.8665
8	0.1185	0.8815	0.1560	0.8440	0.1548	0.8452	0.1156	0.8844	0.1095	0.8905	0.1228	0.8772
9	0.0998	0.9002	0.1453	0.8547	0.1441	0.8559	0.0977	0.9023	0.0978	0.9022	0.1095	0.8905
10	0.0917	0.9083	0.1448	0.8552	0.1438	0.8562	0.0899	0.9101	0.0866	0.9134	0.1041	0.8959
11	0.0881	0.9119	0.1445	0.8555	0.1433	0.8567	0.0864	0.9136	0.0867	0.9133	0.1039	0.8961
12	0.0736	0.9264	0.1344	0.8656	0.1335	0.8665	0.0715	0.9285	0.0718	0.9282	0.0901	0.9099
13	0.0702	0.9298	0.1320	0.8680	0.1310	0.8690	0.0667	0.9333	0.0676	0.9324	0.0848	0.9152
14	0.0622	0.9378	0.1305	0.8695	0.1294	0.8706	0.0589	0.9411	0.0603	0.9397	0.0829	0.9171
15	0.0565	0.9435	0.1246	0.8754	0.1237	0.8762	0.0531	0.9469	0.0548	0.9452	0.0785	0.9215
16	0.0522	0.9478	0.1235	0.8765	0.1228	0.8772	0.0490	0.9510	0.0514	0.9486	0.0743	0.9257
17	0.0492	0.9508	0.1243	0.8757	0.1229	0.8771	0.0473	0.9527	0.0508	0.9493	0.0763	0.9237
18	0.0448	0.9552	0.1239	0.8761	0.1227	0.8773	0.0418	0.9582	0.0482	0.9518	0.0721	0.9279
19	0.0414	0.9586	0.1181	0.8819	0.1172	0.8828	0.0386	0.9614	0.0448	0.9552	0.0704	0.9296
20	0.0381	0.9619	0.1176	0.8824	0.1161	0.8839	0.0345	0.9655	0.0408	0.9592	0.0671	0.9329
21	0.0330	0.9670	0.1178	0.8822	0.1162	0.8838	0.0303	0.9697	0.0394	0.9606	0.0664	0.9336
22	0.0322	0.9678	0.1149	0.8851	0.1140	0.8860	0.0306	0.9694	0.0383	0.9617	0.0640	0.9360
23	0.0333	0.9667	0.1202	0.8798	0.1191	0.8809	0.0305	0.9695	0.0389	0.9611	0.0666	0.9334
24	0.0304	0.9696	0.1231	0.8769	0.1219	0.8781	0.0276	0.9724	0.0383	0.9617	0.0684	0.9316
25	0.0273	0.9727	0.1147	0.8853	0.1136	0.8864	0.0253	0.9747	0.0352	0.9648	0.0656	0.9344
26	0.0261	0.9739	0.1184	0.8816	0.1171	0.8829	0.0235	0.9765	0.0353	0.9647	0.0647	0.9353
27	0.0268	0.9732	0.1195	0.8805	0.1180	0.8820	0.0246	0.9754	0.0365	0.9635	0.0650	0.9350
28	0.0256	0.9744	0.1188	0.8812	0.1171	0.8829	0.0231	0.9769	0.0348	0.9652	0.0650	0.9350
29	0.0246	0.9754	0.1180	0.8820	0.1170	0.8830	0.0224	0.9776	0.0316	0.9684	0.0637	0.9363
30	0.0237	0.9763	0.1135	0.8865	0.1122	0.8878	0.0212	0.9788	0.0323	0.9677	0.0612	0.9388

## Data Availability

The data presented in this study are available on request from the corresponding author due to ongoing related research and data use agreements that limit immediate public sharing.
